# La estructura de los hogares de las personas mayores en América Latina y el Caribe

**DOI:** 10.26633/RPSP.2021.115

**Published:** 2021-09-30

**Authors:** Albert Esteve, Pilar Zueras

**Affiliations:** 1 Centro de Estudios Demográficos (CED-CERCA) Barcelona España Centro de Estudios Demográficos (CED-CERCA), Barcelona, España.; 2 Institute for Social and Economic Research, University of Essex Reino Unido Institute for Social and Economic Research, University of Essex, Reino Unido.

**Keywords:** Hogares, personas mayores, América Latina, Región del Caribe, Housing, aged, Latin America, Caribbean Region, Habitação, idoso, América Latina, Região do Caribe

## Abstract

**Objetivo.:**

Analizar las pautas de convivencia de la población de 60 años o más que reside en hogares privados en 23 países de América Latina y el Caribe.

**Métodos.:**

Estudio transversal realizado con base en los microdatos censales más recientes disponibles en Integrated Public Use Microdata Series (IPUMS)-International, la mayoría de ellos correspondientes a la ronda censal de 2010. Se calcularon y se compararon, para cada país y por sexo, el número medio de convivientes, su distribución por edad y las relaciones de parentesco que se establecen entre ellos. Se compararon, por país y por sexo, el promedio de convivientes en función del nivel de escolaridad y del estado civil.

**Resultados.:**

El promedio del número de personas con las que convive la gente mayor difiere entre países. Oscila entre dos personas en países como Argentina, Puerto Rico y Uruguay, y cuatro o más personas en países como Honduras y Nicaragua. Esta diferencia resulta de la mayor o menor presencia de personas jóvenes, hijos y otros familiares en el hogar. El número de convivientes disminuye con el mayor nivel de escolaridad, salvo en Cuba y en Puerto Rico, donde no se observan diferencias. En general, las mujeres mayores viven en hogares con menos personas que los hombres, aunque no es el caso de las personas solteras o divorciadas.

**Conclusiones.:**

La convivencia con hijos y otros familiares es habitual en la Región. Las diferencias entre países y por nivel educativo muestran que la familia juega un papel importante en la protección social de la vejez en los países menos desarrollados y en los grupos menos escolarizados.

## Abstract (en)

**Objective.:**

Analyze cohabitation patterns in the population over 60 years of age living in private households in 23 countries in Latin America and the Caribbean.

**Methods.:**

Cross-sectional study based on the most recently available census microdata from the Integrated Public Use Microdata Series (IPUMS, International), corresponding mainly to the 2010 census. Average number of household members, age distribution, and family relationships were calculated and compared for each country and by sex. The average number of household members was compared, by country and by sex, in relation to level of schooling and marital status.

**Results.:**

The average number of people that older people live with differs between countries, ranging from two or less in countries such as Argentina, Puerto Rico, and Uruguay, to four or more in countries such as Honduras and Nicaragua. This difference depends on a greater or lesser presence of young adults, children, and other family members in the household. The number of household members declines with a higher level of schooling, except in Cuba and Puerto Rico, where no differences are observed. In general, older women live in households with fewer people than men, although this is not the case for unmarried or divorced people.

**Conclusions.:**

In the Region, it is common for older persons to live with children and other family members. The differences between countries and by educational level show that the family plays an important role in social protection of the elderly in less developed countries and in the least educated groups.

## RESUMO

**Objetivo.:**

Analisar os padrões de convivência da população de 60 anos ou mais que reside em moradias particulares em 23 países da América Latina e do Caribe.

**Métodos.:**

Estudo transversal realizado com base nos microdados censitários mais recentes disponíveis no *Integrated Public Use Microdata Series (IPUMS)-International*, na maior parte correspondente à etapa censitária de 2010. O número médio de coabitantes, sua distribuição por idade e as relações de parentesco estabelecidas entre eles foram calculados e comparados para cada país e por sexo. A média de coabitantes em função do nível de escolaridade e do estado civil foi comparada por país e por sexo.

**Resultados.:**

A média do número de pessoas com quem os idosos convivem difere entre países. Oscila entre 2 pessoas em países como Argentina, Porto Rico e Uruguai e 4 ou mais pessoas em países como Honduras e Nicarágua. Essa diferença resulta da maior ou menor presença de jovens, filhos e outros familiares em casa. Quanto maior o nível de escolaridade, menor o número de coabitantes, exceto em Cuba e em Porto Rico, onde não são observadas diferenças. Em geral, as mulheres idosas vivem em moradias com menos pessoas que os homens, embora não seja o caso das pessoas solteiras ou divorciadas.

**Conclusões.:**

A convivência com filhos e outros familiares é habitual na Região. As diferenças entre países e por nível educacional mostram que a família desempenha um papel importante na proteção social da velhice nos países menos desenvolvidos e nos grupos menos escolarizados.

La familia sigue siendo la principal responsable y proveedora del bienestar de las personas mayores en América Latina y el Caribe (ALC) (1). En 2020, las personas de 60 años o más representaban el 13,0% de la población de la Región. Su peso relativo y absoluto se duplicará en las próximas tres décadas, según las proyecciones de Naciones Unidas (2). Estas generaciones se beneficiaron de importantes mejoras en la supervivencia experimentadas desde mediados del siglo XX, resultado del progreso médico y la reducción de la mortalidad por enfermedades infecciosas (3). A pesar de los diferentes perfiles de la transición demográfica y epidemiológica en ALC, en algunos países la transición avanza a notable velocidad (3-6). El sobreenvejecimiento de la población mayor es aún moderado y heterogéneo, con Argentina, Barbados, Chile, Cuba y Uruguay en los primeros lugares, y Belice, Bolivia, Guatemala, Haití, Honduras y Nicaragua en los últimos (1). Pero la discapacidad está presente en distinto grado entre la población de 60 años o más. En 2010, el 45% de estas personas tenía algún tipo de discapacidad en República Dominicana y Uruguay, y más del 60% en Brasil, mientras que en Ecuador y Venezuela se mantenía en el 20%. El envejecimiento poblacional trae asociados retos sociales y de cuidado que, a diferente ritmo, afecta a todos los países. Algunos de ellos carecen de las estructuras institucionales necesarias para apoyar a esta creciente población y su potencial demanda de cuidados. En ausencia de un sistema de protección social a la vejez, el hogar juega un papel todavía más importante en el bienestar de las personas mayores (1,6,7).

La mayoría de las personas mayores en América Latina reside en hogares privados con más de una persona (8) de su red de parentesco. Los hogares son una unidad básica de funcionamiento de cualquier sociedad (9). Desempeñan funciones esenciales en la crianza y socialización de los hijos, en el cuidado de las personas mayores y de las personas dependientes y en la distribución de recursos económicos entre sus miembros. El tamaño y la estructura de los hogares viene condicionado por factores demográficos, culturales y materiales (10).

Entre los factores demográficos, la fecundidad y la longevidad son los más importantes. La fecundidad determina la mayor o menor presencia de hijos en el hogar, y la longevidad condiciona la supervivencia de los individuos y, en consecuencia, la coexistencia de varias generaciones y el número de años que potencialmente podemos convivir con nuestras parejas, padres, hermanos, y otros familiares (11,12). Pero la disponibilidad de parentesco no es suficiente para entender la estructura de los hogares. Cuando los hijos llegan a edades adultas, las opciones residenciales se diversifican. Esta diversidad es fruto de la interacción entre factores culturales y materiales (13). Los sistemas familiares están moldeados por factores culturales e históricos que establecen las normas de funcionamiento de la formación de la familia, incluida la elección del cónyuge, la herencia, y la formación de hogares (14,15). La familia nuclear promueve la emancipación temprana de sus jóvenes y la formación de hogares independientes. En las sociedades con fuertes lazos familiares, predomina la convivencia intergeneracional y los hogares extensos. En el origen de la familia extensa está la necesidad de proteger el legado familiar, controlar la reproducción y proporcionar cuidado a las personas mayores (15). Ante las explicaciones de corte cultural, suelen contraponerse aquellas que enfatizan los aspectos materiales (16,17). La disponibilidad de recursos económicos y materiales facilita la vida independiente de los adultos. La viabilidad de los hogares depende de su solvencia económica. Sin un sistema de protección social o medios económicos suficientes, las personas mayores que no generan recursos propios suelen depender de sus hijos o familiares. En este sentido, en ALC, la importancia relativa de las transferencias privadas (entre familiares) respecto a las públicas es superior a la de los países occidentales (18). El 64% del total de transferencias son privadas en comparación con el 41% en los países occidentales. Cuando los sistemas públicos son débiles, la gente mayor está obligada a continuar trabajando y depender de sus familiares (19).

América Latina es una región muy diversa en cuanto a su estructura y dinámica familiar (20). La diversidad se observa entre y dentro de los países, por regiones y grupos sociales. Siglos de mestizaje, dominación colonial, informalidad en el mercado laboral y desigualdad económica han tenido una influencia notable en la dinámica familiar. Como resultado de todo ello, la familia en ALC presenta algunas particularidades. La más destacada es la presencia histórica de la unión libre, especialmente entre las poblaciones más vulnerables y desfavorecidas (21-23). Las uniones libres se forman a edades más tempranas, tienen hijos más pronto y son menos duraderas que los matrimonios. La disolución de estas uniones está detrás del elevado porcentaje de madres solteras que hay en la Región. El 70% de estas madres vive en hogares extensos (24) con presencia de adultos mayores, en general sus progenitores. La familia extensa ejerce su papel protector en todas las etapas de la vida. Sin este papel no se entendería por qué los jóvenes siguen formando uniones y teniendo hijos a edades relativamente tempranas (25,26). El hogar extenso en ALC protege más que controla a sus individuos. Vivir en solitario es una opción poco frecuente en ALC a todas las edades (1,8). La proporción de personas que viven solas aumenta a edades avanzadas, pero se mantiene entre el 5 y 20% en todos los países de ALC, con contadas excepciones. Estos valores apenas difieren por sexo, especialmente cuando se controla el estado conyugal (8). El escaso desarrollo y cobertura de los sistemas de pensiones o protección a las personas mayores en la mayoría de los países es un factor contribuyente (1,6). La presencia de hogares extensos o compuestos es común en varios países, y más en estratos sociales bajos (27) y en las personas mayores. En 2010, un tercio o más residía en hogares extensos o compuestos en Argentina, Brasil y México (7).

Estos elementos son relevantes para entender la estructura de los hogares de las personas mayores en ALC, pero las diferencias entre países son importantes. Hay países más ricos que otros. La cobertura pública de pensiones para la gente mayor es también desigual (28). Los países más pobres de la Región se encuentran en Centroamérica (con la excepción de Costa Rica) y los más ricos, en el cono Sur. A su vez, hay países como Brasil, Colombia o México que son muy diversos. Dar cuenta de esta diversidad subnacional queda fuera de los objetivos y del alcance de este artículo. Sin embargo, falta un estudio que examine la estructura de los hogares de la gente mayor en relación con variables sociodemográficas comparables para entender la diversidad entre el mayor número de países de ALC.

El objetivo de este artículo es analizar las pautas de convivencia de la población de 60 años o más que reside en hogares privados en 23 países de ALC y hacer una comparación sobre la base de los datos más recientes que disponemos de la Región. El primer objetivo es estudiar el tamaño y la estructura de los hogares donde residen las personas mayores. El tamaño está dado por el número de personas que conviven en un mismo hogar, y la estructura refleja la composición por edad y las relaciones de parentesco que se establecen entre los convivientes. El segundo objetivo es comparar las pautas de convivencia de hombres y mujeres en estos países, en función del nivel de escolaridad y el estado civil.

## MATERIALES Y MÉTODOS

Este estudio es un análisis transversal realizado a partir de los microdatos anonimizados de los censos de población de 23 países de ALC: Argentina, Bolivia (Estado Plurinacional de), Brasil, Chile, Colombia, Costa Rica, Cuba, Ecuador, El Salvador, Guatemala, Haití, Honduras, Jamaica, México, Nicaragua, Panamá, Paraguay, Perú, Puerto Rico, República Dominicana, Trinidad y Tabago, Uruguay y Venezuela (República Bolivariana de). La mayoría de los datos corresponden a la ronda censal de 2010, a excepción de Colombia y Nicaragua (2005), y Chile, Guatemala, Haití, Honduras, Jamaica, Paraguay y Venezuela (2001-2003). Estos datos proceden de las muestras más recientes de microdatos censales disponibles en la base de datos de Integrated Public Use Microdata Series (IPUMS)-International (29). La IPUMS proporciona datos armonizados procedentes de censos y encuestas para más de 102 países de todo el mundo, desde la década de los 60 a la actualidad. De todas las regiones del mundo, ALC es la que tiene mayor presencia en IPUMS. Se trata de muestras representativas a escala nacional con una densidad muestral que incluye entre el 5 y el 10% de la población. Los microdatos individuales están organizados por hogares, lo que permite analizar variables de composición de hogar en relación con variables sociodemográficas individuales.

Se seleccionó la población mayor de 60 años, a la que nos referimos como personas mayores, y se analizaron los hogares donde residen. En 2010, este grupo de edad representaba el 10% de la Región, en comparación con 4,5% de mayores de 70 años y 1,4% de mayores de 80 años (2). Tras un análisis exploratorio, se comprobó que los resultados obtenidos no varían de manera sustancial en los grupos de edad más avanzada, salvo en algunos países menos poblados y con menor proporción de población mayor, que presentan la aleatoriedad propia de muestras pequeñas. Se considera la población de 60 años o más con el fin de proporcionar resultados consistentes del máximo número de países dentro de la Región. Además, este grupo de edad es de uso frecuente en los datos de Naciones Unidas y en abundantes estudios que analizan aspectos relacionados con el envejecimiento poblacional y sus retos en ALC. El mayor tamaño muestral corresponde a Uruguay (N = 20 697) y Argentina (N = 18 491) y el menor, a Bolivia (N = 9 486) y Puerto Rico (N = 9 256). Este depende del volumen de población y del avance del envejecimiento en cada país (5), así como de la densidad muestral disponible en IPUMS.

### Variables

Para caracterizar los hogares de las personas mayores, se consideraron tres dimensiones: el número medio de convivientes, la distribución por edad de estos convivientes y las relaciones de parentesco que se establecen entre ellos. Se agrupó la edad de los convivientes en menores de 20 años, de 20 a 59 años y de 60 años o más. Respecto al parentesco, se distinguieron los siguientes tipos de relación: hijo o hija, padre o madre, hermano o hermana, otros familiares y personas sin relación de parentesco. Dentro de otros familiares estarían los nietos y las nietas, las parejas de los hijos y las hijas y otros. Esta distinción es posible gracias a las variables que proporciona IPUMS sobre las relaciones intrafamiliares entre los miembros de un mismo hogar. Los resultados se presentan desagregados por sexo, en función del nivel de escolaridad y el estado civil o conyugal. Se agrupó el nivel de escolaridad en tres categorías: menos de primaria, primaria completa y secundaria completa. Se definieron cuatro categorías para el estado civil: soltero o soltera, casado, casada o en unión libre, separado, separada o divorciado o divorciada, y viudo o viuda. Esta variable no está disponible para Argentina.

### Análisis estadístico

Para cada país y sexo, se calculó el número medio de convivientes en los hogares de la gente mayor. La persona de referencia no está incluida en el cálculo, es preciso añadir una persona para obtener el tamaño medio del hogar. Se utilizaron técnicas de visualización para comparar entre países el tamaño promedio del hogar y la distribución media de las personas convivientes en los grupos de edad considerados. De la misma manera, se compararon el tamaño promedio del hogar y la distribución media de la relación de parentesco de los convivientes. Se presentan gráficas de tendencia por nivel de escolaridad y paneles de diagramas de puntos por estado civil. Para el procesamiento de la información se utilizó MS Excel^®^ y Rstudiov1.3.1093^®^. Los resultados han sido ponderados según los pesos proporcionados en la muestra. No se calcularon los intervalos de confianza porque el tamaño de la muestra y el carácter agregado de los indicadores no los hace necesarios.

La protección de datos de los individuos está garantizada al utilizar fuentes anonimizadas y agrupadas por países, y no fue necesario someter el estudio a un comité de ética.

## RESULTADOS

### Composición de los hogares por edades y relación de parentesco

**FIGURA 1.**
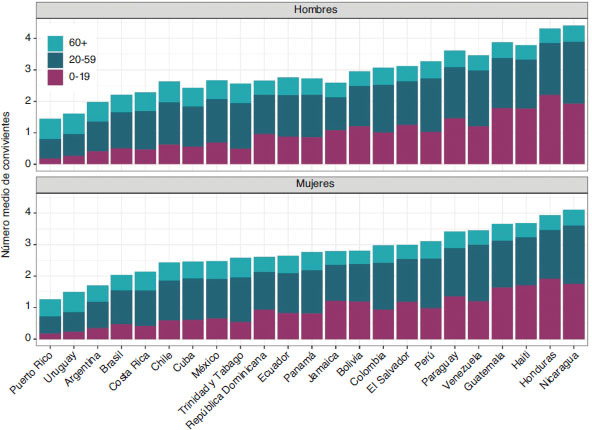
Número medio y edad de las personas que conviven con personas mayores (de 60 años o más) en hogares privados en América Latina, por sexo

En la figura 1 se observa muestra que no existen diferencias reseñables entre hombres y mujeres en cuanto al *ranking* de los países ni tampoco en cuanto al número medio de convivientes, aunque bien es cierto que las mujeres mayores conviven con menos personas que los hombres en la mayoría de países, a excepción de Ecuador, México y Panamá, en los que no hay diferencias. El promedio de la diferencia entre hombres y mujeres es de 0,12 personas a favor de los primeros.

El número medio de personas con las que convive la gente mayor oscila entre las 2 o menos personas en países como Argentina, Puerto Rico (forma parte de los Estados Unidos de América) y Uruguay, y 4 o más personas en países como Honduras y Nicaragua. La mayoría de los países andinos (Colombia, Perú y Venezuela) y los de Centroamérica se encuentran en la franja alta de la distribución. México y los países del Caribe suelen tener valores por debajo de 3. En términos absolutos, el número medio de convivientes de la misma edad es muy similar en todos los países. La cifra promedio oscila entre 0,52 y 0,66 personas. La diferencia entre países viene determinada por la mayor o menor presencia de personas más jóvenes. Entre 60% y 88% de las personas que conviven con personas mayores tienen menos de 60 años. Entre estas, de 23% a 57% son personas menores de 20 años. La presencia de niños sugiere la existencia de hogares de tres generaciones. Prueba de ello es que existe una fuerte correlación entre el número de convivientes de 0 a 19 años y el de convivientes de 20 a 59.

***Fuente:*** elaboración propia a partir de las muestras de IPUMS de microdatos censales de la ronda censal de 2010 para 23 países de América Latina y el Caribe.

**FIGURA 2.**
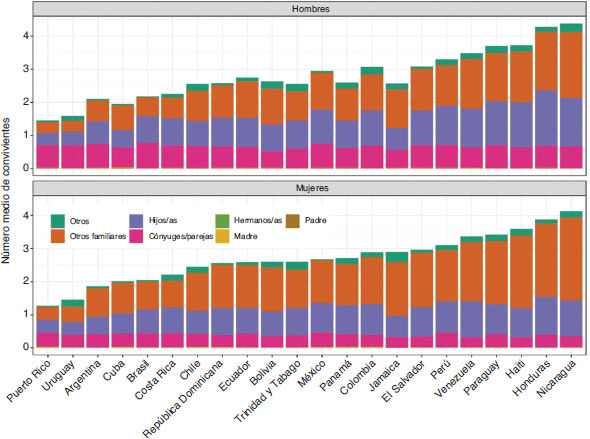
Número medio y relación de parentesco las personas que conviven con personas mayores (de 60 años o más) en hogares privados en América Latina, por sexo

***Fuente:***elaboración propia a partir de las muestras de IPUMS de microdatos censales de la ronda censal de 2010 para 23 países de América Latina y el Caribe.

**FIGURA 3.**
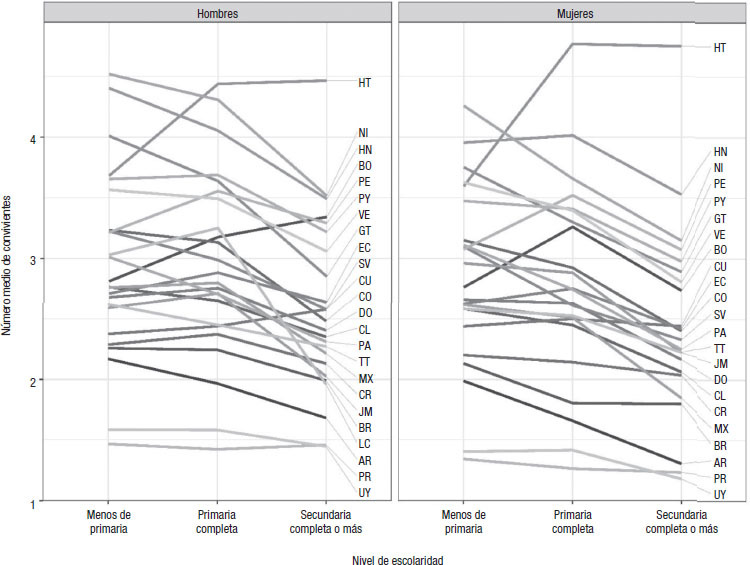
Número medio de convivientes con personas mayores (de 60 años o más) por nivel de escolaridad en hogares privados en América Latina, por sexo

***Fuente:*** elaboración propia a partir de las muestras de IPUMS de microdatos censales de la ronda censal de 2010 para 23 países de América Latina y el Caribe.

La presencia de personas sin relación de parentesco es reducida (figura 2). El promedio de todos los países es de 0,12 personas, y los valores mínimos y máximos oscilan entre 0,03 (Puerto Rico) y 0,3 (Jamaica) personas. De menor a mayor, según sea el valor promedio para toda la Región, las relaciones de parentesco más comunes son la pareja (0,51), los hijos e hijas (0,86) y otros familiares (1,22). Los hombres mayores suelen convivir con sus parejas en mayor número que las mujeres. Por países, las principales diferencias se observan en relación con la mayor o menor presencia de otros familiares. La diferencia entre el valor mínimo y el máximo supera las dos personas. En un extremo están las personas mayores de Puerto Rico, que son las que menos conviven con otros familiares (0,33 personas en el caso de los hombres). En el extremo opuesto, estarían las mujeres mayores de Nicaragua que son las que más conviven con otros familiares; 2,49 personas en promedio. En función del país, los hijos e hijas aportan entre 0,36 y 1,66 convivientes de media por hogar si se toman los valores mínimos y máximos con independencia del sexo.

### Promedio de convivientes en función del nivel educativo y del estado civil

El número de personas convivientes disminuye con el nivel de escolaridad en 18 de los 23 países analizados tanto para hombres como para mujeres (figura 3). En Haití se observa la relación opuesta, también en los hogares de hombres en Bolivia. En Perú no hay una tendencia clara. Y en Cuba y en Puerto Rico, no se observa relación entre el nivel de escolaridad y las pautas de convivencia. La diferencia en el número medio de convivientes entre el nivel bajo de escolaridad y el nivel alto alcanza como máximo una persona de diferencia. Por lo general, el *ranking* de países se mantiene en todos los niveles de escolaridad.

**FIGURA 4.**
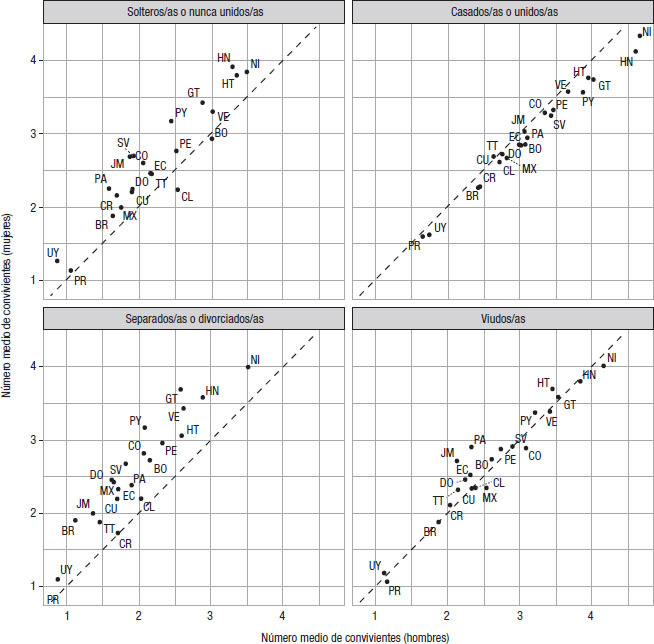
Diferencias entre hombres y mujeres en el número medio de convivientes con personas mayores (de 60 años o más) por estado civil en hogares privados en América Latina, por sexo

En la figura 4 se observa que, en promedio, los hogares de las personas mayores casadas o unidas son más grandes que los de las personas viudas, las solteras y las separadas. Entre las personas casadas y las viudas, apenas hay diferencias por sexo en el número medio de convivientes. Sin embargo, los hombres solteros o los divorciados y separados residen en hogares más pequeños que las mujeres en la misma situación. La diferencia entre unas y otros puede alcanzar una persona de diferencia.

***Fuente:*** elaboración propia a partir de las muestras de IPUMS de microdatos censales de la ronda censal de 2010 para 22 países de América Latina y el Caribe. Nota: la variable no está disponible para Argentina.

## DISCUSIÓN

El análisis de las pautas de convivencia de la población de 60 años o más muestra que el número medio de personas con las que convive la gente mayor en ALC varía poco por sexo, pero mucho por países. En la mayoría de los países analizados, las personas mayores conviven con 2 o más personas, que suelen ser más jóvenes. La mayor presencia de personas más jóvenes, hijos y otros familiares marca la diferencia entre los países. En muchos de ellos, los niveles de convivencia intergeneracional son elevados, a juzgar por la presencia de hijos y otros parientes (probablemente, nietos y nietas) en el hogar, mientras la presencia de miembros no familiares es más bien modesta. El tamaño del hogar de las personas mayores tiene relación con su nivel educativo: cuanto mayor es su nivel de escolaridad menor el número de convivientes. Con respecto al estado civil, el número medio de convivientes no varía en función del sexo para la población casada y la viuda, que reside en hogares más grandes que el resto. Los hombres divorciados y separados residen en hogares más pequeños que las mujeres en la misma situación. Sobre estas tendencias generalizables al conjunto de la Región, existen variaciones importantes entre países. Puerto Rico y Uruguay destacan por ser los países donde el promedio de convivientes es menor. La gente mayor en los países del Cono Sur suele vivir en hogares más pequeños y menos complejos que la gente mayor de los países andinos y Centroamérica (con la clara excepción de Costa Rica). Las pautas de convivencia de las personas mayores en Brasil y México son parecidas a las del Cono Sur. La mayor heterogeneidad se encuentra en los países del Caribe.

En línea con estudios previos, los resultados muestran la importancia de los hogares extensos en América Latina en todas las etapas de la vida (6,30), pero también las variaciones en las organizaciones familiares de las personas mayores por nivel educativo y estado civil y según su composición por edades y parentesco de los convivientes. Las organizaciones familiares de las personas mayores en ALC son mucho más densas que en Europa, Estados Unidos, Canadá, y algunos países más ricos de Asia (como Japón) y África (como Sudáfrica), y a menudo involucran la presencia de dos generaciones adultas (1,8,9,30).

Algunos aspectos que preocupan en Europa, como la soledad de los mayores en hogares privados no generan aún la misma preocupación en América Latina (8). Ahora bien, es probable que esta situación refleje la vulnerabilidad de las familias en la Región y la necesidad de que las generaciones se protejan mutuamente (6). La informalidad de las uniones, la inestabilidad laboral, o la escasa protección social ante la vejez o el desempleo promueven la solidaridad entre familiares (24,30); los hogares extensos son una forma de expresarla. El hecho de que la convivencia intergeneracional de los mayores sea menos frecuente en aquellos países de ALC con un índice de desarrollo humano y económico más elevado apoyaría esta interpretación (28,31). También lo haría el hecho de que, por lo general, el número medio de convivientes es menor allí donde la gente tiene mayor nivel de escolaridad. El nivel de escolaridad es también muy desigual. Mientras en algunos países, como Bolivia, Brasil, Ecuador, México y República Dominicana, cerca de 25% o más de la población de 60 años o más era analfabeta, esa proporción era menor de 5% en Argentina o Uruguay (1). Se desconoce la capacidad y la solvencia económica de las personas adultas que comparten hogar y si el hecho de que vivan juntas viene determinado únicamente por la necesidad. En esta línea, un estudio reciente muestra que, en algunos países, el aporte económico de las personas mayores al ingreso de los hogares multigeneracionales es notable (6).

Las limitaciones de este estudio tienen que ver con la naturaleza de los datos. La ausencia de información generalizada sobre los ingresos, la dirección de las transferencias dentro del hogar, la dependencia y la protección social limita el alcance del análisis y la comparación entre países muy heterogéneos. Al tratarse de datos censales, la heterogeneidad de variables incluidas disminuye la capacidad de un análisis comparativo en profundidad. El objetivo de incorporar el mayor número de países posibles reduce el número de variables comparables. Sin embargo, esta elección también conlleva fortalezas. La inclusión de 23 países ha permitido constatar con datos empíricos la importancia de la convivencia intergeneracional de la gente mayor en ALC, así como la gran diversidad dentro de la Región, y comprobar que el nivel educativo desempeña un papel importante en la independencia residencial de las personas mayores en toda la Región.

## Conclusiones

La población mayor en América Latina y el Caribe está poco aislada en sus hogares. Los niveles de convivencia con hijos y otros familiares son elevados en la Región. La convivencia con otros familiares es menor en los países más ricos y entre las personas mayores con niveles educativos altos. La familia juega un papel importante en el cuidado de los adultos mayores. Las políticas públicas encaminadas a mitigar los efectos del envejecimiento deben tener en cuenta esta realidad con apoyos directos a los hogares.

## Declaración.

Las opiniones expresadas en este manuscrito son únicamente responsabilidad de los autores y no reflejan necesariamente los criterios ni la política de la *Revista Panamericana de Salud Pública* o de la Organización Panamericana de la Salud.
